# *Chlamydia pneumoniae* CopD Translocator Protein Plays a Critical Role in Type III Secretion (T3S) and Infection

**DOI:** 10.1371/journal.pone.0099315

**Published:** 2014-06-24

**Authors:** David C. Bulir, Daniel A. Waltho, Christopher B. Stone, Kenneth A. Mwawasi, Jordan C. Nelson, James B. Mahony

**Affiliations:** M. G. DeGroote Institute for Infectious Disease Research, Faculty of Health Sciences and Department of Pathology and Molecular Medicine, McMaster University, and Father Sean O'Sullivan Research Centre, St. Joseph's Healthcare, Hamilton, Ontario, Canada; Oregon State University, United States of America

## Abstract

Pathogenic Gram-negative bacteria use type III secretion (T3S) to inject effector proteins into the host cell to create appropriate conditions for infection and intracellular replication. *Chlamydia spp.* are believed to use T3S to infect their host cell, and the translocator proteins are an essential component of this system. *Chlamydia pneumoniae* contains genes encoding two sets of translocator proteins; CopB and CopD, and CopB2 and CopD2. In this study, we identified novel interactions between CopD and three type III secretion proteins; namely, CopN, CdsN, and CdsF. We identified a CopD putative chaperone binding motif, PxLxxP, within the N-terminal region (CopD amino acids 120–125), which was necessary for interaction with its putative chaperone LcrH_1. Using size exclusion chromatography, we showed that CopD and LcrH_1 formed higher order structures in solution with CopD and LcrH_1 binding in a ratio of 1∶1, which is unique for T3SS translocator proteins. Lastly, we showed that antibodies to CopD reduced *C. pneumoniae* infectivity by >95%. Collectively, this data suggests that CopD plays a critical role in pathogenesis and likely functions as a hydrophobic translocator of the type III secretion system in *Chlamydia pneumoniae*.

## Introduction

*Chlamydia* species are obligate intracellular pathogens with a unique biphasic lifecycle initiated by the attachment of the metabolically quiescent elementary body (EB) to the host cell and subsequent invasion into a plasma-membrane derived vacuole termed an inclusion body [1]. Inside the inclusion, EB transform into metabolically active reticulate bodies (RB) that remain associated with the inclusion membrane [1]. *Chlamydia* RB are thought to interact with the host cell cytoplasm across the inclusion membrane using the type III secretion (T3S) injectisome. *Chlamydiae* are capable of commandeering host cell pathways to acquire lipids, cholesterol, and other nutrients crucial for growth and replication and some of these functions may be mediated by T3S. RB continue to replicate until an unknown signal triggers differentiation into EB, which temporally coincides with detachment of the RB and the T3S injectisome from the inclusion membrane [2]. *Chlamydiae* then exit the cell through either lysis or a packaged release mechanism termed extrusion [1]. The complete replication cycle takes approximately 48–72 hours depending on the species.

T3S is a virulence mechanism used by several Gram-negative bacteria, including *Yersinia, E. coli*, and *Salmonella* to inject effector proteins from the bacterial cytoplasm directly into the host cell [3], [4]. The T3SS consists of 20 to 25 components, all of which form a functional T3S injectisome [3], [4]. The needle filament protein (YscF in *Yersnia*) extends from the bacterial outer membrane into the extracellular matrix, and houses a distal needle-tip complex. This needle-tip complex contains the needle-tip protein (LcrV orthologs) and the translocators (YopB and YopB orthologs), which are involved in sensing the host cell and initiating secretion [5]. Upon host cell contact, a signal is transmitted to the inner membrane effector recognition complex, which consists of several membrane proteins including an ATPase [6]. The ATPase binds effector-chaperone complexes, dissociating the effector from their cognate chaperone followed by unfolding to facilitate their passage through the injectisome [6]. The translocators present at the tip of the complex initiate pore formation in the host cell in preparation for effector secretion.

In contrast to other chaperones that play a role in protein folding and assembly of macro-molecular structures, T3S chaperones maintain proteins in a secretion competent state. Type I chaperones are subdivided into two categories; type IA and type IB [7]. Type IA chaperones are known to bind to one specific effector protein, whereas type IB chaperones are capable of binding to more than one effector protein, such as Slc1 [7]. Type I chaperones share some similar biochemical properties, including a low molecular weight (∼20 kDa), an acidic isoelectric point (pI), and interacting with cognate effectors as a homo-dimer [7]. Furthermore, these chaperones bind conserved chaperone binding domains (CBD) that encompass hydrophobic regions on their binding partner, usually located at the N-terminus of their cognate effector. Type II chaperones are chaperones for hydrophobic, oligomeric translocator proteins, and include SycD (*Y. enterocolitica*), LcrH (*Y. pestis*), SicA (*Salmonella spp.*), IpgC (*Shigella spp.*), Scc2/Scc3 (Specific chlamydia chaperone) (*Chlamydia trachomatis*), and Cpn0811 (LcrH_1), and Cpn1021 (LcrH_2) (*C. pneumoniae*) [4], [7], [8]. Amino acid sequence analysis has shown the presence of tetratricopeptide repeat (TPR) domains present in all type II chaperones, including *Chlamydia* [9]. TPR domains are a common structural motif for protein-protein interactions in chaperones, which are present in eukaryotic chaperones such as Hsp70 and Hsp90 [9], [10]. Furthermore, type II chaperones maintain the hydrophobic translocators in a secretion competent state, and prevent premature hetero- or homo-oligomerization of the translocators in the bacterial cytosol by masking the oligomerization domain [10].

The translocator proteins form a translocon pore in the host cell membrane and are broadly divided into hydrophobic and hydrophilic translocators [10]. The hydrophilic translocator is the sensor protein, which detects the host cell and triggers secretion of effectors through an as yet unknown mechanism [10]. This sensing mechanism has been linked with cholesterol-rich lipid rafts, and other small molecules in the host cell membrane. The hydrophobic translocators are believed to be early effector proteins, consisting of the major hydrophobic translocator (YopB in *Yersinia*) and the minor hydrophobic translocator (YopD in *Yersinia*). In *Yersinia*, YopB and YopD form the translocon pore in the host cell membrane, which is instrumental in docking the injectisome to the membrane via the needle filament protein and allowing translocation of effector proteins into the host cell cytoplasm. The hydrophobic translocators in *Chlamydia* have been poorly characterized. Genetic analysis of *C. pneumoniae* has identified two putative pairs of translocators; namely, *cpn0809* and *cpn0808* (CopB and CopD, respectively) and *cpn1020* and *cpn1019* (CopB2 and CopD2, respectively) [8]. Cpn0808, annotated as CopD, is believed to be the minor hydrophobic translocator [2], [11]. The minor hydrophobic translocator (YopD orthologs) in other bacteria interact with other key components of the T3SS, including the filament protein, the major hydrophobic translocator, the ATPase, and the plug protein [10], [12], [13]. Initial work on the translocators by Fields *et. al.* indicated that the translocator proteins, CopB and CopD, from *Chlamydia trachomatis* may be type III secreted, as evidenced by a heterologous secretion system. Furthermore, they suggest that the SycD orthologous proteins, Scc2 and Scc3, may function as translocator chaperones [11]. More recent work by Chellas-Gery *et. al.* has expanded our knowledge of CopB, and implicated it as a potential translocator protein from *Chlamydia trachomatis* [14]. These translocator proteins have dedicated chaperones (LcrH_1 and LcrH_2) that maintain them in a partially unfolded, secretion-competent state until they are translocated to the needle-tip complex.

In this report, we characterize *cpn0808* (CopD), a putative translocator protein of *C. pneumoniae* to assess its role in chlamydial T3S. We identified novel interactions between CopD and the type III secretion proteins CopN, CdsN, and CdsF. We identified a specific N-terminal region, CopD_1–157_, containing a putative chaperone binding motif, PxLxxP, which is required for interaction with the LcrH_1 chaperone. Collectively, this data supports a role for CopD as a hydrophobic translocator of the T3SS in *Chlamydia pneumoniae*.

## Methods and Materials

### Cloning

All T3S genes were cloned from *C. pneumoniae* CWL029 (VR1310, ATCC) using genomic DNA as template. Since full-length CopD was toxic to *E. coli*, fragments excluding transmembrane domains based on TMpred software were cloned. The following genes were cloned into the pDONR201 vector with *attB*-containing primers via the Gateway cloning system (Invitrogen) (subscript denotes amino acid number): *cdsN*, *lcrH_1, copN, cdsF, copD_1–137_, copD_158–206_, copD_227–444_, copD_1–157_, ^P120A^copD_1–157_, ^L122A^copD_1–157_, and ^P125A^copD_1–157_*. The pDONR201 vector containing the respective gene were then cloned into pDEST17 (N-Terminal 6×His-tag), pDEST15 (N-Terminal GST-tag) and pDEST-HisMBP (N-Terminal 6×His-Maltose Binding Protein-tag) for protein expression. Full length *copD* and *lcrH_1* was amplified with appropriate restriction sites for cloning into MCS1 (N-Terminal 6×His-tag) and MCS2 (C-Terminal S-tag), respectively, of the pET-DUET vector (Novagen). All constructs were verified by sequencing at the MOBIX Laboratory at McMaster University.

### Protein Expression and Purification

All expression constructs were transformed into *E. coli* Rosetta pLysS strains to minimize protein expression prior to induction with isopropyl β-D galactosidase (IPTG). Briefly, 6 L of LB containing 100 µg/mL ampicillin was inoculated with 1∶50 dilution of an overnight culture. The culture was then grown at 37°C with shaking at 250 RPM until an absorbance at 600 nm of 0.600 was reached. Prior to induction with 0.2 mM IPTG, the cultures were cooled to 16°C on ice. After induction, the cultures were left incubating at room temperature with shaking at 250 RPM for 3 hours. After induction, the bacteria were pelleted at 8000× *g* in a Sorval RC-5B centrifuge at 4°C. The bacterial pellets were washed once with cold phosphate-buffered saline (PBS) and then resuspended in either Nickel A (20 mM TRIS-HCl pH 7.0, 500 mM KCl, 0.03% LDAO, 10 mM imidazole, 10% glycerol) or PBS, depending on the downstream application. The bacteria were then subjected to sonication and pelleted at 50000× *g* to isolate the soluble protein. Polyhistidine-tagged proteins were purified using fast protein liquid chromatography (FPLC) using a Ni-NTA His-Trap HP column (GE Healthcare), and washed with 5%,10%, and 15% Nickel B before elution in 100% Nickel B (20 mM TRIS-HCl pH 7.0, 500 mM KCl, 0.03% LDAO, 300 mM imidazole, 10% glycerol). Prior to size exclusion chromatography, proteins were buffer exchanged into phosphate buffered saline with 0.03% LDAO.

### Glutathione-S-transferase (GST) pull-down assay

Glutathione-agarose beads (Sigma) were swollen in distilled water for 2 hours at room temperature, and then washed with PBS. GST-tagged proteins were bound to 1 mL of glutathione-agarose beads for two hours at 4°C while rocking. The GST-bound agarose beads were centrifuged at 3000× *g* for 5 minutes to remove the supernatant and then blocked with 5% bovine serum albumin (BSA) in PBS+0.1% TWEEN-20 overnight at 4°C while on a rocking platform. Blocked beads (50 µL) were mixed with 1 mL of *E. coli* lysates containing our overexpressed His-tagged protein, and left rocking at 4°C for 2 hours. The glutathione-agarose beads were then centrifuged at 16000× *g* for 10 seconds and the supernatant was removed, and the pellet washed with high salt wash buffer (500 mM KCl, 20 mM TRIS-HCl pH 7.0, 0.1% Triton X-100). Washing was repeated seven times. The last supernatant wash was collected, trichloroacetic acid (TCA) precipitated, and analyzed using sodium dodecyl sulfate polyacrylamide gel electrophoresis (SDS-PAGE) and Western blot to confirm that His-tagged protein was removed. The glutathione-agarose beads were then resuspended in 30 µL of Laemmli buffer and heated at 90°C for 20 minutes. The samples were then analysed by SDS-PAGE and Western blot analysis using a mouse anti-His antibody (Sigma).

### Bioinformatics

To identify orthologous sequences, CopD was subjected to BLASTP (Basic Local Alignment Search Tool Protein) and PSI-BLAST using the default parameters, and excluding *Chlamydia* spp. from the search. Structural characteristics within CopD were analyzed using the TMpred tool to identify transmembrane domains, using a minimum transmembrane window of 17 and maximum of 33. COILS online software was used to predict the presence of coiled-coil domains within CopD, using MTIDK scoring matrices, and weighting for positions a & d [14].

### Size exclusion chromatography

Full length His-CopD and LcrH_1-S-tag (300 µL, 4.5 mg/mL) mixture was subjected to size exclusion chromatography in PBS+0.03% LDAO (Sigma) on a Superdex S200 10/300 GL gel filtration column (Amersham Biosciences, Piscataway, New Jersey) at 0.5 mL/min [15]. Elution fractions of 1 mL were collected at a flow rate of 0.5 mL/min, with each fraction analyzed via an anti-His and anti-S-tag Western blot. Size prediction of elution complexes was performed after the column was standardized using a LMW and HMW gel filtration standard kit (GE Life Sciences) [15].

### Inhibition of *C. pneumoniae* infection

A polyclonal antibody to a 15 amino acid peptide (SSKGEKSEKSGKSKC) was produced and obtained from GenScript (New Jersey). *C. pneumoniae* was pre-incubated for 2 hours at 37°C with various dilutions of affinity purified CopD antibody, control antibody, or pre-immune sera. Infection was performed as previously described by Johnson *et. al.* [16]. After 72 hours, chlamydial inclusions were stained with the Pathfinder *Chlamydia* detection reagent (BioRad). Multiple fields of view were visualized and percent reduction of infection was calculated compared to control infection. Statistical significance was calculated using a Student's t-test.

## Results

### Bioinformatic analysis of CopD

**Figure 1:**
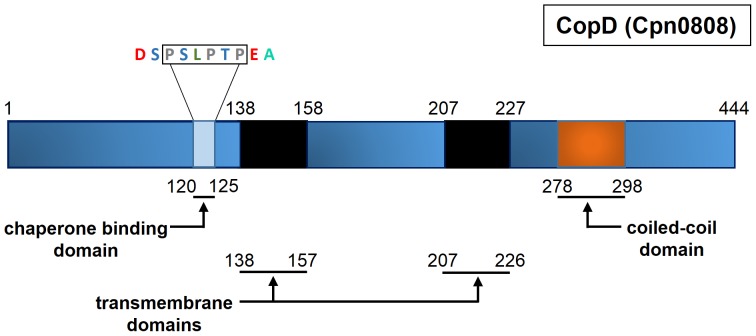
Topographic overview of structural prediction of CopD. Solid black regions represent transmembrane domains. Orange block represents a predicted coiled-coil domain in the C-terminus of the protein. Light blue depicts predicted Chaperone Binding Domain (CBD) located from amino acids 120–125.

Translocator proteins are highly conserved across bacterial species because of their critical role in T3S [3], [4]. We used a bioinformatic approach to examine similarities between translocators from *Chlamydia* and other bacterial species. Despite the inability to identify an orthologous protein through PSI-BLAST and BLASTP, CopD contains many structural and sequence characteristics consistent with a translocator protein. Cpn0808 is a 444 amino acid protein consisting of two *in silico* predicted transmembrane domains and one coiled-coil domain. The TMpred server was applied for transmembrane domain prediction with a minimum and maximum hydrophobic window of 17 and 33 amino acids, respectively. Consistent with orthologous minor hydrophobic translocators, *viz.* YopD or IpaC, Cpn0808 contains two transmembrane domains consisting of amino acids 138–157 and 207–226 [10], [14]. Furthermore, the coiled-coil domain prediction program (COILS) identified a 21 amino acid coiled-coil domain spanning amino acids 278–298 (Figure 1). It is known that translocator chaperones bind within the N-terminus of typical translocator proteins [17]. Previous studies on bacterial T3SS translocators have identified a conserved chaperone binding motif; namely, PxLxxP [17]. We identified that same motif within Cpn0808 (PSLPTP) at amino acid 120, which could function as the chaperone binding site. Collectively, the bioinformatic analysis of CopD suggests that CopD is a hydrophobic translocator protein of the type III secretion system.

### Interaction of CopD with Type III Components

**Figure 2:**
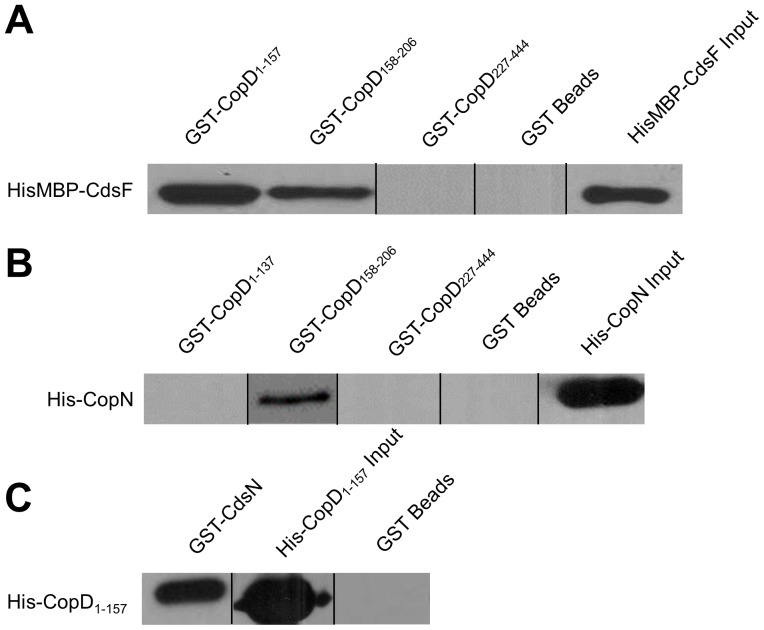
Chlamydia Outer Protein (Cop) D Interacts with T3S proteins CdsF, CopN, and CdsN. GST-CopD_1–157_ or GST-CopD_158–206_ bound to glutathione-agarose beads (bait) pulled HisMBP-CdsF (prey) out of an *E. coli* lysate in the presence of a high salt wash buffer (500 mM NaCl). Furthermore, GST-CopD_158–206_ pulled His-CopN out of an *E. coli* lysate in the presence of a high salt wash buffer. Lastly, GST-CdsN pulled His-CopD_1–157_ out of an *E. coli* lysate in the presence of a high salt wash buffer.

To investigate protein interactions of CopD with other T3SS proteins we performed *in vitro* interaction studies using glutathione-S-transferase (GST) pull-down assays. We identified novel interactions between Cpn0808 and CopN, CdsF, CdsN. These assays were performed under high salt conditions (500 mM NaCl) in the presence of 0.1% Triton X-100 to eliminate non-specific interactions. Due to the difficulty of working with full-length CopD in *E. coli*, fragments of CopD were cloned and used for pull down assays. We identified an interaction between CopD_1–157_, CopD_158–206_ and the needle protein, CdsF (Figure 2A). In orthologous systems, the minor hydrophobic translocator is known to interact with the plug protein, and we found that amino acids 158–206 of CopD interacts with CopN (Figure 2B) [18]. Furthermore, the N-terminal fragment of CopD_1–137_ interacted with the T3SS ATPase, CdsN (Figure 2C). The observation that CopD interacts with three key proteins of the T3S apparatus suggests that CopD plays an important role in chlamydial T3S.

### Interaction of LcrH_1 and CopD

**Figure 3:**
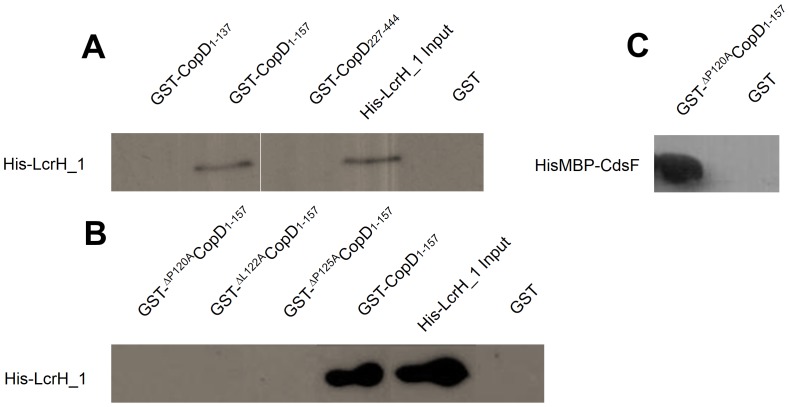
LcrH_1 (*Cpn0811*) interacts with CopD at amino acids 120–125. Recombinant LcrH_1 interacted with amino acids 1–157 of CopD. CopD mutants were created through overlapping PCR to create ^P120A^CopD_1–157_, ^L122A^CopD_1–157_, and ^P125A^CopD_1–157_. Mutations at the conserved amino acids within the predicted chaperone binding domain disrupted the interaction between CopD_1–157_ and the chaperone LcrH_1, but not other identified interactions.

Analysis of YopD orthologs indicates the presence of a conserved CBD containing a PxLxxP motif [17]. Bioinformatic analysis of CopD revealed a PxLxxP motif extending from amino acid 120 to 125. Before determining the essential amino acids in this PxLxxP motif, we showed that LcrH_1 interacted with full-length CopD using a co-expression and purification in the presence of high salt (500 mM KCl) and detergent (0.03% LDAO) (data not shown). To identify the specific binding region of LcrH_1 on CopD, fragments of CopD were made to identify the interacting domains. GST-CopD_1–157_ interacted with His-LcrH_1 in the presence of high salt (500 mM KCl) and detergent (0.1% Triton X-100), but did not interact with GST-CopD_1–137_, or GST-CopD_227–444_ (Figure 3A). Together, these results suggest that amino acids 1–157 of CopD contains a required domain for interaction with the putative chaperone LcrH_1.

To identify the necessity of the chaperone binding motif, an alanine walkthrough of the conserved amino acids in the CopD PxLxxP motif was performed to examine the requirement of the three conserved amino acids for binding LcrH_1. To ensure that the CBD variants retained the ability to interact with previously identified binding partners, we performed a GST pull down assay of GST-^ΔP120A^CopD_1–157_ and CdsF. Figure 3C demonstrates that the variants retained the ability to interact with CdsF. His-LcrH_1 interacted with GST-CopD_1–157_, but not with GST-^ΔP120A^CopD_1–157_, GST-^ΔL122A^CopD_1–157_, and GST-^ΔP125A^CopD_1–157_ (Figure 3B). These results suggest that amino acids 120,122 and 125 of CopD are required for interaction with LcrH_1. However, in the absence of being able to show that each variant does not result in a major conformational change in the molecule thereby precluding interaction with CdsF, we cannot say with certainty that these amino acids are essential for CopD binding with its partners.

### Oligomerization of CopD

**Figure 4:**
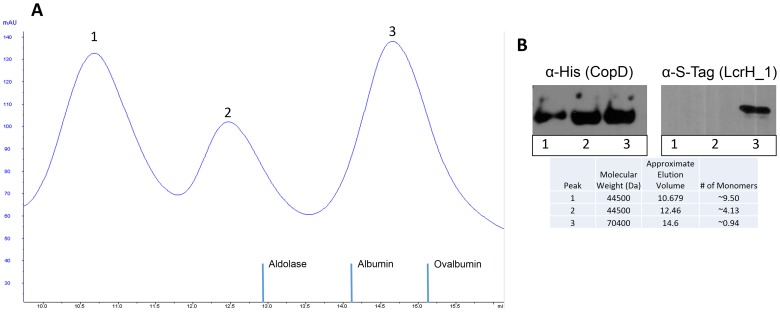
Interaction of CopD and LcrH_1 occurs in a 1∶1 ratio assessed by size exclusion chromatography of co-expressed proteins. CopD and LcrH_1 were co-expressed, purified, and subjected to size exclusion chromatography. Elution peaks were analyzed by Western Blot using anti-His antibody for CopD and anti-S-tag for LcrH_1. Panel A shows size exclusion chromatography elution peaks. Panel B shows the Western Blot result for the three peaks, and the corresponding the number of CopD monomers present in peaks 1, 2, and 3.

Since translocators of other bacterial secretion systems are capable of forming higher order structures including homo- or hetero-oligomers, we explored whether CopD could form homo-oligomers. To determine whether CopD forms higher-order structures, His-CopD was co-expressed with LcrH_1-S-tag and subjected to size-exclusion chromatography in the presence of 0.03% LDAO. His-CopD eluted from the column with peaks corresponding to a decamer, tetramer and monomer, as determined by Western Blot using antibodies to the poly-histidine tag. When the membrane was probed with an anti-S-tag antibody, LcrH_1 was detected only in the fraction containing monomeric CopD (Figure 4). Similar results were obtained when His-CopD and LcrH_1-S-tag were subjected to size exclusion chromatography in the presence of 0.1% Triton X-100 (data not shown). This data indicates that CopD is capable of forming higher order structures in solution and that LcrH_1 binds only to monomeric CopD [19], [20].

### Neutralization of *C. pneumoniae* infection with antibodies to CopB and CopD

**Figure 5:**
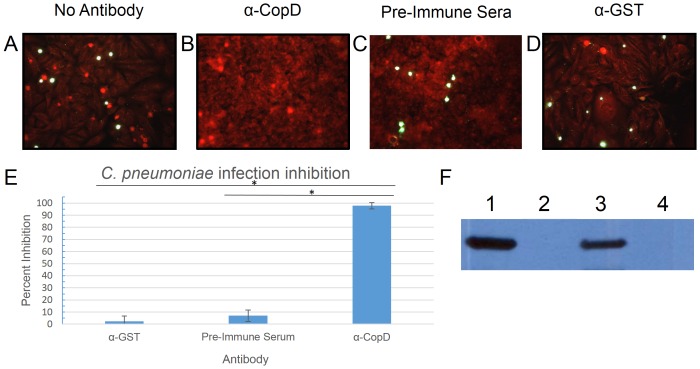
Inhibition of *Chlamydia pneumoniae* with CopD antibodies. Panels A–D show inhibition assay results performed with either no antibody (A), CopD antibody (B), pre-immune sera (C), or control antibody (α-GST) (D). Panel E shows the degree of inhibition by of CopD antibodies compared to control antibodies. Chlamydial inclusions are stained green, while HeLa cells are stained red by Evan's blue counterstain. Panel F demonstrates reactivity of anti-CopD with (1) *C. pneumonia* infected HeLa cell lysate, (2) uninfected HeLa cell lysate, (3) recombinant GST-CopD_1–157_ produced in *E. coli*, and (4) recombinant GST produced in *E. coli*. Experiments were performed in triplicate. Error bars represent 2 standard deviations. Images represent random fields of view. * = P<0.0001.

Since T3S translocators are believed to be surface exposed and antibodies generated against these antigens could potentially block invasion of host cells, we investigated the possibility that antibodies to CopD might inhibit *C. pneumoniae* infection. We used affinity purified polyclonal antibodies raised against an N-terminal CopD peptide (SSKGEKSEKSGKSKC) to investigate whether these antibodies could block infection. The CopD 16-mer within the N-terminal region was predicted to be immunogenic since it is hydrophilic with a net positive charge. Based on these characteristics, this sequence of amino acids is most likely solvent exposed and capable of interacting with an antibody. We first showed that the anti-CopD antibodies reacted with both recombinant and native CopD from *C. pneumoniae* by Western blot analysis prior to performing infection inhibition assays (Figure 5, Panel F). Pre-incubation of *C. pneumoniae* EB with affinity purified anti-CopD antibody inhibited infection by 98%, as compared to control antibody, as determined by IF staining of inclusion bodies (Figure 5). This suggested that CopD may be either surface exposed or secreted and that it plays a critical role in infection of cells.

## Discussion

T3S systems have been well-characterized for several bacterial systems, but chlamydial T3S remains poorly characterized, and little is known about the chlamydial translocator proteins. In this study, we characterized the putative minor hydrophobic translocator CopD (Cpn0808) of *C. pneumoniae*. We showed that CopD interacted with other *Chlamydia* type III secretion proteins including, CopN, CdsF, and CdsN. Bioinformatic analysis revealed that CopD contains structural features consistent with other minor hydrophobic translocators, including two transmembrane domains and a coiled-coil domain. In addition, CopD interacted with its putative chaperone, LcrH_1, first, by an essential chaperone binding motif of PxLxxP at amino acids 120–125, and second, via a predicted transmembrane domain at amino acids 138–157. We also showed that LcrH_1 interacts with only monomeric CopD in a 1∶1 ratio and not tetrameric or decameric CopD, as evidenced by size exclusion chromatography. Finally, we show that polyclonal antibodies directed against an N-terminal epitope of CopD inhibited chlamydial infection. Collectively, these findings are consistent with CopD functioning as a hydrophobic translocator of the *C. pneumoniae* T3S apparatus.

**Figure 6:**
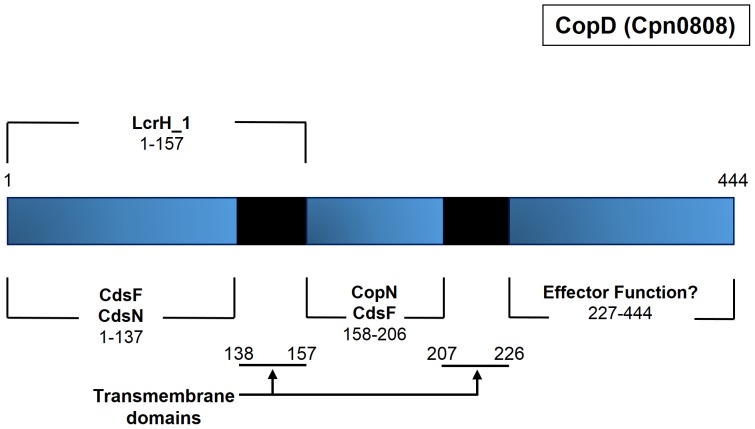
Summary of proposed interactions between CopD and type III secretion proteins. Interactions identified through Glutathione-S-Transferase pull down assays mapped to a topographic overview of CopD.

Since T3S knock-outs cannot be made in *Chlamydia* it is not possible to unequivocally demonstrate the role of individual T3S components. We have used *in vitro* protein interactions to demonstrate interactions between CopD and three other T3S proteins. CdsN, the ATPase of the *Chlamydia* T3SS, plays a key role in substrate selection and mediates effector-chaperone disassociation prior to secretion, allowing effectors to be translocated through the injectisome. We demonstrated that the N-terminal fragment of CopD interacted with CdsN. This suggests that CopD is delivered to the base of the needle apparatus, possibly associated with its putative chaperone, allowing CdsN to dissociate the effector-chaperone complex to initiate secretion. The filament protein, CdsF, and its orthologs in other bacteria, form the needle of the injectisome and is believed to play a role in facilitating the insertion of translocators into the host cell membrane [21]. We have demonstrated that CopD interacts with CdsF, and have identified two specific regions of CopD, *viz.* CopD_1–157_ and CopD_158–206_ that facilitate this interaction. In *Yersinia spp.*, the plug protein, YopN, has been shown to interact with YopD [18]. We explored this interaction in *C. pneumoniae* using a GST pull down assay and confirmed the interaction between CopD_158–206_ and CopN (Figure S1). A summary of the interactions identified in this study appear in Figure 6. It is interesting to note that all of the protein interactions that we have identified occur within the N-terminus of CopD, and we have not identified any proteins that interact within the C-terminus of CopD. Based on orthologous T3S systems, we believe that this C-terminal domain of CopD is involved in homo-oligomerization, membrane interaction, or other effector functions consistent with orthologs as suggested by other T3S systems [22]–[27].

A bioinformatic analysis of CopD demonstrated predicted structural and sequence motifs found in other hydrophobic translocator proteins [22], [28], [29]. PSI-BLAST or BLASTP failed to identify a CopD orthologous protein outside of the *Chlamydiales* order, but revealed that CopD contains two predicted transmembrane domains (amino acids 138–157 and 207–226) and a predicted coiled-coil domain (amino acids 207–226), characteristic structures of other hydrophobic translocators. The toxicity of full-length CopD in *E. coli* could be due to the presence of two transmembrane domains of CopD, which may insert into the bacterial membrane, forming homo-oligomers and thus disrupting normal membrane function and causing cell death. This phenomenon has been observed with other translocator proteins when they are not co-expressed with their putative chaperone [30]. Since expression of full-length CopD alone yielded insoluble protein, CopD was co-expressed with its putative chaperone, LcrH_1. In principle SEC allows for the estimation of molecular weight from the elution volume. Since Western blot results indicate the presence of both CopD and LcrH_1 in the third elution peak, it is most likely that CopD (44.5 kDa) and LcrH_1 (25.9 kDa) are present as a hetero-dimer with a combined molecular weight of 70 kDa. If CopD or LcrH_1 formed homo-dimers they would be expected to elute in a different volume. In addition, any LcrH_1 in the mixture must be complexed with His-CopD since the complex was purified on Ni-NTA agarose. Our SEC data suggesting that CopD and LcrH_1 interact in a 1∶1 molar ratio, is consistent with a 1∶1 molar ratio shown for IpaB and IpgC in *Shigella* [30], [31]. Another difference found in *Chlamydia* T3SS is that they possess two sets of predicted translocators, despite only having a single T3SS [2], [8], [14]. Furthermore, we found that CopD formed higher order structures in the absence of LcrH_1, corresponding to decamers and tetramers. Together, this data suggests that the role of LcrH_1 is to maintain CopD in a monomeric state, preventing homo-oligomerization of CopD prior to secretion. The presence of a conserved chaperone binding motif, PxLxxP, within the N-terminal region supports the possibility of CopD being a translocator protein. Furthermore, the coiled-coil domain located near the C-terminus may function to bring the C-terminal domain of the protein in contact with the membrane to mediate its effector function, such as actin polymerization. Further studies are required to elucidate the function of this C-terminal region. Collectively, the bioinformatics data supports that CopD may serve as a hydrophobic translocator for chlamydial T3S.

It is well known that LcrH_1 orthologs interact within the N-terminal region of translocator proteins. In this report, we show that LcrH_1 interacts with the N-terminus of CopD (amino acids 1–157). A hydrophobic sequence of amino acids, 138–157, of CopD appear to play a critical role in the interaction with LcrH_1, as GST-CopD_1–157_ but not CopD_1–138_ interacted with His-LcrH_1. In other T3S systems, LcrH_1 is believed to function by masking hydrophobic amino acids and preventing premature homo- and hetero-oligomerization prior to secretion, corroborating the importance of amino acids 138–157 for interaction.

In addition to amino acids 138–157, the interaction is dependent on amino acids 120–125 within the PxLxxP motif, suggestive of two possible binding domains. A mutational analysis of the conserved CBD motif, PxLxxP (P120→A, L122→A, P125→A), of CopD was undertaken to examine the role of these conserved amino acids. An alanine walk through of the conserved amino acids of the PxLxxP motif was performed. Substitution of each of the three amino acids in the PxLxxP motif (P120→A, L122→A, P125→A) abolished the interaction with LcrH_1, indicating the essential role of these amino acids in the CopD/LcrH_1 interaction. To our knowledge, this is the first demonstration of conserved amino acids within the predicted CBD motif of a chlamydial translocator protein. In *C. pneumoniae*, two proteins, CopB and CopD, are believed to form the translocon pore in the host cell. Like CopD, CopB (Cpn0809) is in a contiguous operon with its predicted chaperone, LcrH_1. It is not surprising that starting at amino acid 166, CopB contains a putative chaperone binding domain motif of PxLxxP. However, further studies are required to identify the functionality of this motif in CopB. Examining the predicted orthologous proteins to CopD in the *Chlamydiaceae* family reveals similarities in the predicted chaperone binding domain (Table 1). Of the members of the *Chlamydiaceae, C. pneumoniae, C. psittaci, C. pecorum, and C. abortus* all contain the same PxLxxP, but interestingly, *C. trachomatis* and *C. muridarum* contain an AxLxxP motif. The variability in the motif in *C. trachomatis* is not surprising since the orthologs in *C. trachomatis* and *C. muridarum* have only 56% and 54% sequence identity, respectively, which is the lowest sequence identity among the sequences examined from the *Chlamydiaceae* family. The high sequence identity (>70%) between LcrH_1 orthologs within the *Chlamydiaceae* family, and the conserved CBD domain, or variant thereof, located within the N-terminal region of the CopD orthologs suggests that the CopD-LcrH_1 interaction may be conserved within the *Chlamydiaceae* family.

**Table 1:** Comparison of putative chaperone binding domains between *Chlamydiaceae* and other T3SS containing Gram-negative bacteria.

	P1		P3			P6	
**CopD (** ***C. pneumoniae*** **)**	**P**	S	**L**	P	T	**P**	**100%**
**CT579 (** ***C. trachomatis*** **)**	**A**	T	**L**	P	S	**P**	**54%**
**CopD1 (** ***C. psittaci CP3*** **)**	**P**	Q	**L**	P	T	**P**	**63%**
**TC_0868 (** ***C. muridarum*** **)**	**A**	S	**L**	P	S	**P**	**56%**
**CPE3_0915 (** ***C. pecorum P787*** **)**	**P**	Q	**L**	P	S	**P**	**58%**
**CAB924 (** ***C. abortus S26/3*** **)**	**P**	Q	**L**	P	T	**P**	**63%**
**YopD (** ***Y. enterocolitica*** **)**	**P**	E	**L**	I	K	**P**	**15%**
**SipC (** ***S. enterica*** **)**	**P**	T	**L**	S	A	**P**	**12%**

Putative chaperone binding domains were identified within the N-terminal regions of orthologous proteins to CopD from *C. pneumoniae*. P1, P3, P6, represent positions 1, 3, and 6, respectively of the PxLxxP motif. Percent identity refers to amino acid sequence identity comparing CopD to full length sequences of orthologous proteins.

We have shown that antibodies to CopD inhibit the ability of *C. pneumoniae* to infect cells, which is consistent with observations seen with antibodies to orthologous translocator proteins in other bacteria including *Shigella* (IpaB and IpaD) and *Yersinia* (YopD) [32]–[34]. Since CopD antibodies are unlikely to enter EBs, neutralization with CopD antibodies suggests that that CopD is either surface exposed or secreted during the infection cycle. This observation is consistent with *Chlamydia* being an obligate intracellular pathogen that is dependent on T3S to infect cells. The production of CopD late during the replication cycle when RB differentiate into infectious EB is also consistent with a role for CopD in T3S and infection [8]. Given the essential nature of the translocator proteins in T3S, these proteins may represent an excellent target for drug development and a vaccine candidate.

## Supporting Information

Figure S1 **Chlamydia Outer Protein (Cop) D Interacts with CopN in a pull down assay.** GST-CopD_158–206_ bound to agarose beads reacted with an *E. coli* lysate over-expressing His-CopN in the presence of a high salt wash buffer (500 mM KCl) (middle lane). His-CopN did not interact with Glutathione-S-Transferase (GST) beads in the absence of CopD (left lane). His-CopN input is shown in right lane. The anti-His antibody was specific and did not react with other proteins.
(TIF)
