# Supplementary figures and images for: Chlamydia pneumoniae CopD Translocator Protein Plays a Critical Role in Type III Secretion (T3S) and Infection
Source: PLoS One. 2014 Jun 24;9(6):e99315. doi: 10.1371/journal.pone.0099315 (PMC4068993; doi:10.1371/journal.pone.0099315)

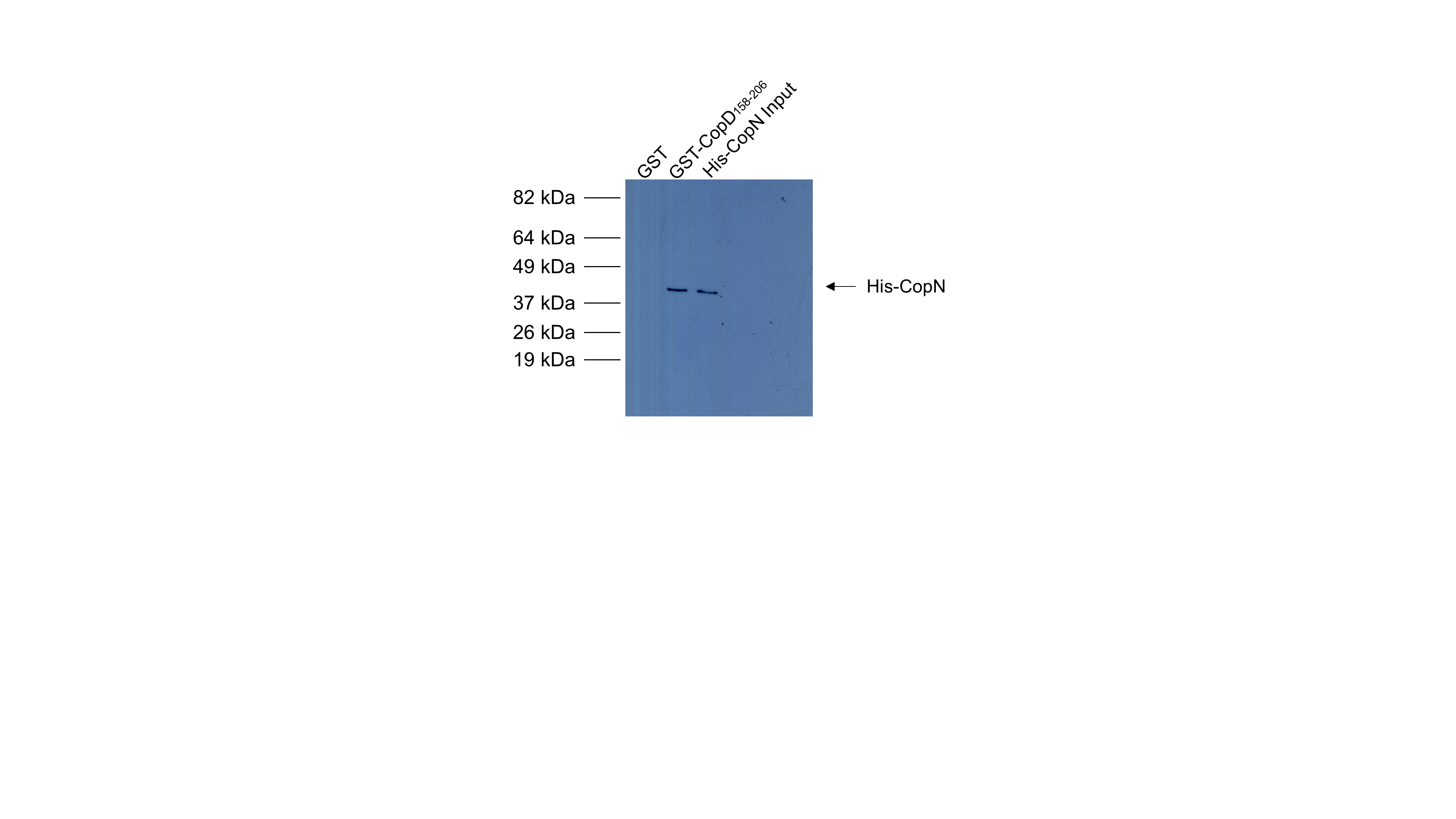

Supplement: Figure S1 — Chlamydia Outer Protein (Cop) D Interacts with CopN in a pull down assay. GST-CopD158–206 bound to agarose beads reacted with an E. coli lysate over-expressing His-CopN in the presence of a high salt wash buffer (500 mM KCl) (middle lane). His-CopN did not interact with Glutathione-S-Transferase (GST) beads in the absence of CopD (left lane). His-CopN input is shown in right lane. The anti-His antibody was specific and did not react with other proteins. (TIF) [file pone.0099315.s001.tif]
